# Assessing synchronous ovarian metastasis in gastric cancer patients using a clinical-radiomics nomogram based on baseline abdominal contrast-enhanced CT: a two-center study

**DOI:** 10.1186/s40644-023-00584-5

**Published:** 2023-07-24

**Authors:** Qian-Wen Zhang, Pan-Pan Yang, Yong-Jun-Yi Gao, Zhi-Hui Li, Yuan Yuan, Si-Jie Li, Shao-Feng Duan, Cheng-Wei Shao, Qiang Hao, Yong Lu, Qi Chen, Fu Shen

**Affiliations:** 1 Department of Radiology, Changhai Hospital, The Navy Medical University, 168 Changhai Road, Shanghai, 200433 China; 2 Department of Emergency, the Eighth Medical Center of Chinese, PLA General Hospital, 17 Heishanhu Rd, Haidian District, Beijing, 100091 China; 3 Department of Radiology, Ruijin Hospital Luwan Branch, Shanghai Jiaotong University School of Medicine, Shanghai, China; 4 GE Healthcare China, Pudong New Town, No.1 Huatuo Road, Shanghai, 210000 China; 5 Department of Health Statistics, The Navy Medical University, Shanghai, 200433 China

**Keywords:** Gastric cancer, Radiomics, CT, Synchronous ovarian metastasis

## Abstract

**Background:**

To build and validate a radiomics nomogram based on preoperative CT scans and clinical data for detecting synchronous ovarian metastasis (SOM) in female gastric cancer (GC) cases.

**Methods:**

Pathologically confirmed GC cases in 2 cohorts were retrospectively enrolled. All cases had presurgical abdominal contrast-enhanced CT and pelvis contrast-enhanced MRI and pathological examinations for any suspicious ovarian lesions detected by MRI. Cohort 1 cases (*n* = 101) were included as the training set. Radiomics features were obtained to develop a radscore. A nomogram combining the radscore and clinical factors was built to detect SOM. The bootstrap method was carried out in cohort 1 as internal validation. External validation was carried out in cohort 2 (*n* = 46). Receiver operating characteristic (ROC) curve analysis, decision curve analysis (DCA) and the confusion matrix were utilized to assess the performances of the radscore, nomogram and subjective evaluation model.

**Results:**

The nomogram, which combined age and the radscore, displayed a higher AUC than the radscore and subjective evaluation (0.910 vs 0.827 vs 0.773) in the training cohort. In the external validation cohort, the nomogram also had a higher AUC than the radscore and subjective evaluation (0.850 vs 0.790 vs 0.675). DCA and the confusion matrix confirmed the nomogram was superior to the radscore in both cohorts.

**Conclusions:**

This pilot study showed that a nomogram model combining the radscore and clinical characteristics is useful in detecting SOM in female GC cases. It may be applied to improve clinical treatment and is superior to subjective evaluation or the radscore alone.

**Supplementary Information:**

The online version contains supplementary material available at 10.1186/s40644-023-00584-5.

## Background

Gastric cancer (GC) is the third deadliest malignancy, with over 1 million newly diagnosed cases and approximately 76,900 deaths in 2020, causing a massive burden worldwide [1, 2]. Distant metastasis is one of the major factors contributing to high mortality in GC. Ovarian metastasis, a unique kind of distant metastasis in females, is not rare in gastric cancer, especially in Asian countries [3]. For example, in Japan, metastases account for almost 20% of all ovarian malignancies, and the most common primary site is the stomach [4]. In clinical studies, the reported incidence of ovarian metastasis ranged from 0.3% to 6.7% in female GC patients. However, according to autopsy studies, an incidence of 33–41% was found for ovarian metastasis [5]. The inconsistency between these clinical and autopsy studies indicated that the incidence of ovarian metastasis might be underestimated in real-world practice.

Ovarian metastasis (OM) in GC patients is correlated with poor prognosis, showing a median overall survival of 11 months [6, 7]. Moreover, synchronous ovarian metastasis (SOM), defined as OM observed within 6 months of the first GC diagnosis, exhibits shorter overall survival compared with metachronous ovarian metastasis [4, 8]. In female GC patients, preoperative detection of SOM is essential for tumor staging and treatment decision making, providing baseline information for individual treatment. When SOM is detected preoperatively, the role of aggressive surgical approach remains controversial, and multidisciplinary team (MDT) discussion is required for individual treatment strategies. Improving outcomes in patients affected by metastatic GC represents an urgent clinical need. Several novel therapies are under investigation, including margetuximab, HER2-targeted therapies, and immunotherapy [9–12].

Precise preoperative diagnosis of SOM is challenging in clinical practice. Indeed, the symptoms of ovarian metastasis are variable and nonspecific. Although ultrasound, CT and MRI are useful for detecting ovarian masses, the imaging features of ovarian metastases sometimes may be atypical and misleading [7]. When occurring unilaterally, ovary metastasis could be hardly differentiated from primary ovarian tumors. Moreover, in some cases, an ovarian mass constitutes the initial sign of cancer. About 7% of ovarian tumors that present as primary ovarian neoplasms are known to be ovarian metastases [13]. In case the possibility of metastatic carcinoma is not considered, treatment options may be adversely affected. On account of management differences and prognostic implications, invasive histopathological approaches such as biopsy and exploratory laparotomy are often required for a confident diagnosis [13].

In the past decades, researchers have focused on the clinical characteristics, treatment methods and prognostic analysis of ovarian metastasis [8–16]. Studies have determined the risk factors for ovarian metastasis in GC patients; however, there have been no reliable models applied to clinical practice so far. Gao et al.reported premenopausal status, tumor invasion depth, number of positive lymph nodes, and no ERβ expression as factors independently predicting metachronous ovarian metastasis [17]. Li et al.constructed a nomogram including age, N stage, Lauren type, signet-ring cell component, estrogen receptor expression, neutrophil/lymphocyte ratio, and serum CA125 for predicting ovarian metastasis in GC, with an area under the curve (AUC) for the model of 0.819 [18]. However, both synchronous and metachronous ovarian metastases were involved in this study, which may not mimic the real-world setting. SOMs need to be detected preoperatively, and a reliable and noninvasive method is required to detect SOM in GC patients.

Recently, several reports have demonstrated radiomics may help radiologists solve tough clinical tasks. By extracting numerous quantitative features from medical images via high-throughput analysis, radiomics could enable radiologists to improve diagnostic accuracy, which eventually would benefit patients. Radiomics-based models have shown a promising value in the detection of occult peritoneal metastasis and lymph node metastasis in GC [19–24].

To our knowledge, a radiomics nomogram for the detection of SOM in GC patients has not been developed. Therefore, the aim of the present work was to build a CT-based radiomics nomogram model for preoperative detection of SOM and to evaluate its clinical application in female GC patients.

## Methods

### Participants

The trial followed the Declaration of Helsinki and had approval from the Ethics Committees of Changhai hospital and Ruijin Hospital Luwan Branch. Due to a retrospective design, signed informed consent was not required.

From January 2019 to March 2022, 174 females with GC detected pathologically at Changhai Hospital were enrolled in this retrospective trial. Inclusion criteria were: (1) gastric adenocarcinoma diagnosed by biopsy or postoperative pathological examination; (2) gastric adenocarcinoma as single focus; (3) both abdominal contrast-enhanced CT examination and pelvis contrast-enhanced MR examination performed at the time of diagnosis; (4) pathological examinations performed for any suspicious ovarian lesions detected by MRI. Exclusion criteria were: (1) local or systemic treatment prior to baseline CT scan (*n* = 46); (2) previously diagnosed or concurrent cancers other than GC (*n* = 3); (3) poor image quality (*n* = 7); (4) metachronous ovarian metastasis (*n* = 12); (5) clinically suspected SOM but not pathologically confirmed (*n* = 5). Therefore, 101 cases were finally included for final analysis as cohort 1. Then, 46 patients meeting the eligible criteria in Ruijin Hospital Luwan Branch between January 2021 and March 2022 were also enrolled as cohort 2.

### Clinicopathologic data

Patient information and clinical findings were retrospectively retrieved from the clinicopathological databases, e.g., age, tumor location (including the upper third, middle third and lower third of the stomach), carcinoembryonic antigen (CEA), carbohydrate antigen 19–9 (CA19-9), carbohydrate antigen 125 (CA125) and carbohydrate antigen 72–4 (CA 724), were recorded at the same time as CT scans (time interval < 2 weeks). All cases were pathologically confirmed as GC, then categorized into 2 groups, including the SOM and no-SOM groups. All pathological SOMs extracted from surgical specimens were confirmed by pathological findings.

### Image acquisition and analysis

Routine contrast-enhanced abdominal CT was performed on a multidetector row CT (MDCT) system (Aquilion, TOSHIBA, Japan; iCT256, PHILIPS, Netherlands) after 4 h of fasting. Supine patients were intravenously injected an iodinated contrast agent at 80–95 ml (Optiray, Liebel-Flarsheim Canada, Canada) at 3.0 or 3.5 ml/s. This was followed by arterial and portal venous phase contrast-enhanced CT after delays of 28 s and 50 s, respectively. CT images were acquired at 120 kV, 100 to 150 mA and 0.5 s rotation time. Contrast-enhanced CT images were reconstructed with the following parameters: field of view, 350 × 350 mm; data matrix, 512 × 512; in-plane spatial resolution, 0.6 mm; axial slice thickness, 5.0 mm; spiral pitch, 1.

Subjective evaluation for SOM was performed by 3 radiologists with systematic training, including QW. Z., PP. Y. and F. S. with 8, 9 and 12 years of experience in CT diagnosis, respectively, who had no knowledge of pathological data. Any discrepancy among them was discussed until an agreement was reached by at least 2 of these experts. The Kappa statistic was used to evaluate interobserver correlation between two given radiologists. Intraclass correlation coefficient (ICC) was determined for evaluating consistency among the three radiologists.

### Image segmentation

The acquired DICOM data (portal venous phase CT scans) were preprocessed with the Artificial Intelligence Kit software (AK, GE Healthcare, China). Images were resampled (using Bspline as the default interpolator) and normalized for subsequent radiomics analysis (using default values). Then, the preprocessed images were imported into the ITK-SNAP software (www.itksnap.org) to manually segment the entire GC tissue layer by layer to obtain the volume of interest (VOI), which reflects the border best fitting the lesion’s area for each GC case. Two radiologists (QW. Z. and PP. Y.) independently repeated the segmentation process in 30 randomly selected patients a week later to analyze observer’s agreement. Finally, all VOIs were imported into the AK software for feature extraction.

### Radiomics feature extraction and reduction

According to the obtained VOIs, 4 categories of features were determined, including first-order feature (voxel intensity distribution on CT images), shape feature (3D properties of the VOIs), texture feature (quantitation of region heterogeneity differences, e.g., gray-level co-occurrence, run length, size zone and neighborhood gray-tone difference matrices) and higher-order feature (transformed first-order data and texture features, including logarithm, exponential, gradient, square, square root, local binary pattern [LBP] and wavelet transformations) groups. Totally 1218 radiomics features were obtained in every patient.

Inter- and intra-observer correlation coefficients (ICCs) were obtained for the assessment of feature robustness. Features with both inter- and intra-observer correlation coefficients above 0.9 were employed to build the model, with outstanding feature reproducibility. To select optimal features associated with SOM, the least absolute shrinkage and selection operator (LASSO) algorithm was utilized (ten-fold cross-validation). The selected features were employed to develop a radscore.

### Nomogram model building and validation

**Fig. 1:**
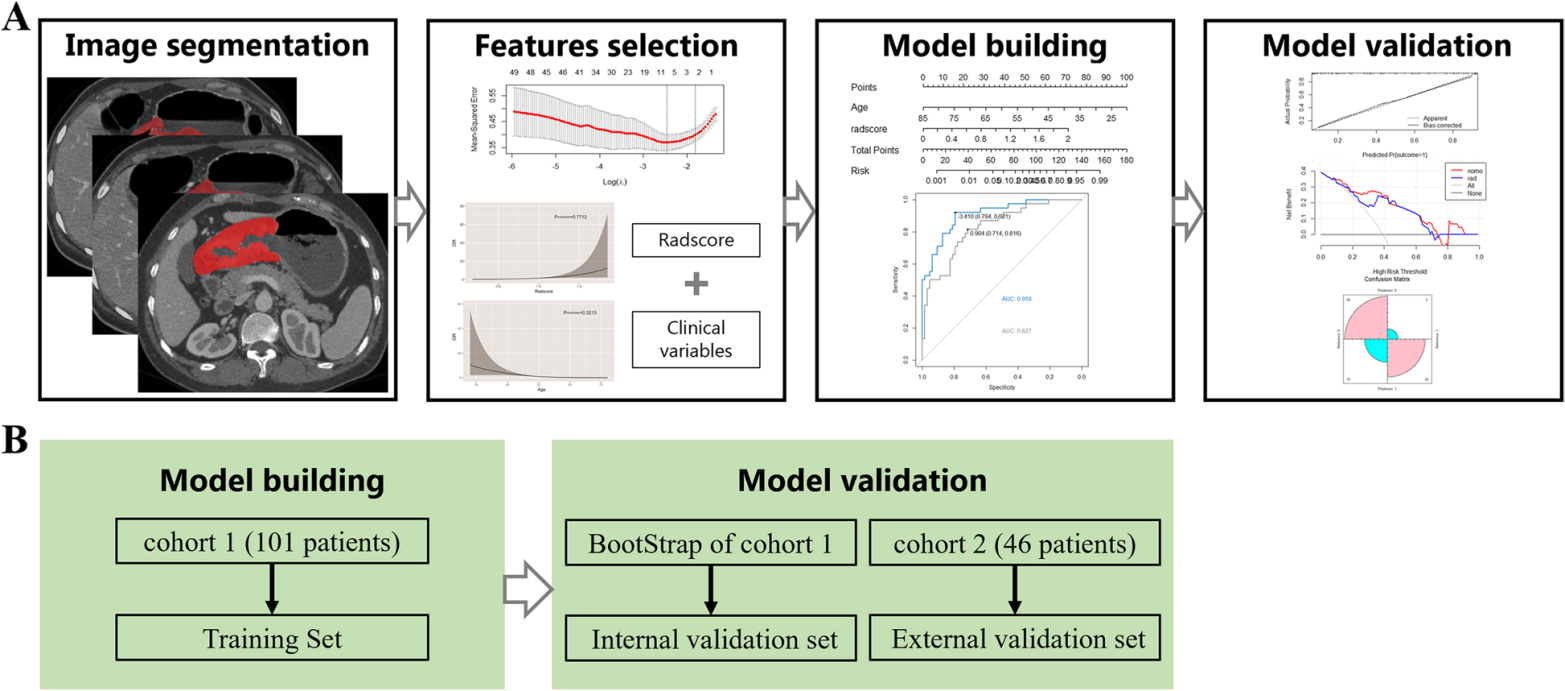
Study flowchart and modeling methods. Study flowchart (**A**) and modeling methods (**B**). Cohort 1, Changhai Hospital; Cohort 2, Ruijin Hospital Luwan

The predictive values of clinical features and the radscore in the detection of SOM were evaluated by univariable logistic regression analysis in the training set (cohort 1). Factors showing *p* < 0.05 were then used to generate a visual nomogram model by multiple factor logistic regression with the stepwise selection method (*p* < 0.05). The bootstrap method (1000 cross-validation) was performed to validate the precision of detection [25] as an internal validation tool in cohort 1. Receiver operating characteristic (ROC) curve analysis was carried out to assess the performances of the radscore, nomogram and subjective evaluation model. Then, the external validation data set (cohort 2) was used for verification. Finally, the models were compared by the DeLong test, and the nomogram’s goodness-of-fit was determined using the Hosmer–Lemeshow test. Decision curve analysis (DCA) and the confusion matrix were utilized to validate clinical benefits. Figure 1 shows the study’s workflow.

### Statistical Analysis

MedCalc 15.2.2 and Python 3.5 were utilized for data analysis. Categorical data were compared by the Pearson chi-square test or Fisher’s exact test, whereas continuous data (mean ± standard deviation) were compared by the Student’s t-test or Mann–Whitney *U* test. Two-sided *P* < 0.05 was deemed to be statistically significant.

## Results

### Patient features

Totally 101 cases were finally enrolled in cohort 1 and 46 patients were included in cohort 2. The two datasets had no marked differences in demographic characteristics (all *P* > 0.05), as shown in Table 1. According to pathological reports, 38/101 (37.6%) cases were SOM in cohort 1, versus 18/46 (39.1%) in cohort 2. Interobserver agreement for the subjective evaluation in both cohorts is shown in Supplemental Table 1.

**Table 1:** Clinicopathological parameters of the examined cohorts

Clinicopathological parameter		Cohort 1 (*n* = 101)	Cohort 2 (*n* = 46)	*P* value
**Menstrual status**				0.343
	**postmenopause**	48 (47.5%)	18 (39.1%)	
	**premenopause**	53 (52.5%)	28 (60.9%)	
**Age (years) **^**a**^		49.762 ± 14.265	54.391 ± 11.174	0.054
**Tumor location**				0.317
	**U**	20 (19.8%)	5 (10.9%)	
	**M**	41 (40.6%)	18 (39.1%)	
	**L**	40 (39.6%)	23 (50.0%)	
**CEA **^**b**^		1.955 (1.110, 3.915)	2.120 (1.110, 3.930)	0.587
**CA125 **^**b**^		16.400 (10.300, 57.200)	16.050 (9.900, 54.300)	0.902
**CA125/CEA **^**b**^		8.389 (4.375, 23.694)	6.503 (2.899, 22.187)	0.939
**CA724 **^**b**^		3.640 (1.480, 7.782)	3.620 (1.580, 7.560)	0.923
**CA19-9 **^**b**^		12.830 (4.900, 70.752)	12.830 (7.130, 53.810)	0.770
**Group**				0.862
	**Without SOM**	63 (62.4%)	28 (60.9%)	
	**With SOM**	38 (37.6%)	18 (39.1%)	

*U* upper third of the stomach, *M* middle third of the stomach, *L* lower third of the stomach, *SOM* synchronous ovarian metastasis

^a^ Mean ± SD

^b^ Median (IQR)

### Radiomics features and selection

After inter- and intra-observer agreement analyses, 921/1218 (75.6%) features showed inter- and intra-observer ICCs ≥ 0.9, and were used for radiomics analysis. Eventually, two features were determined by the LASSO algorithm to be optimal (Supplemental Fig. 1), and a radscore was built as follows:

$$\mathrm{Radscore}=0.0122029783\ast(\mathrm{original\_shape\_Maximum}3\mathrm{DDiameter})+0.0002702978\ast(\mathrm{original\_glrlm\_ShortRunHighGrayLevelEmphasis}).$$

### Nomogram model construction and evaluation

**Fig. 2:**
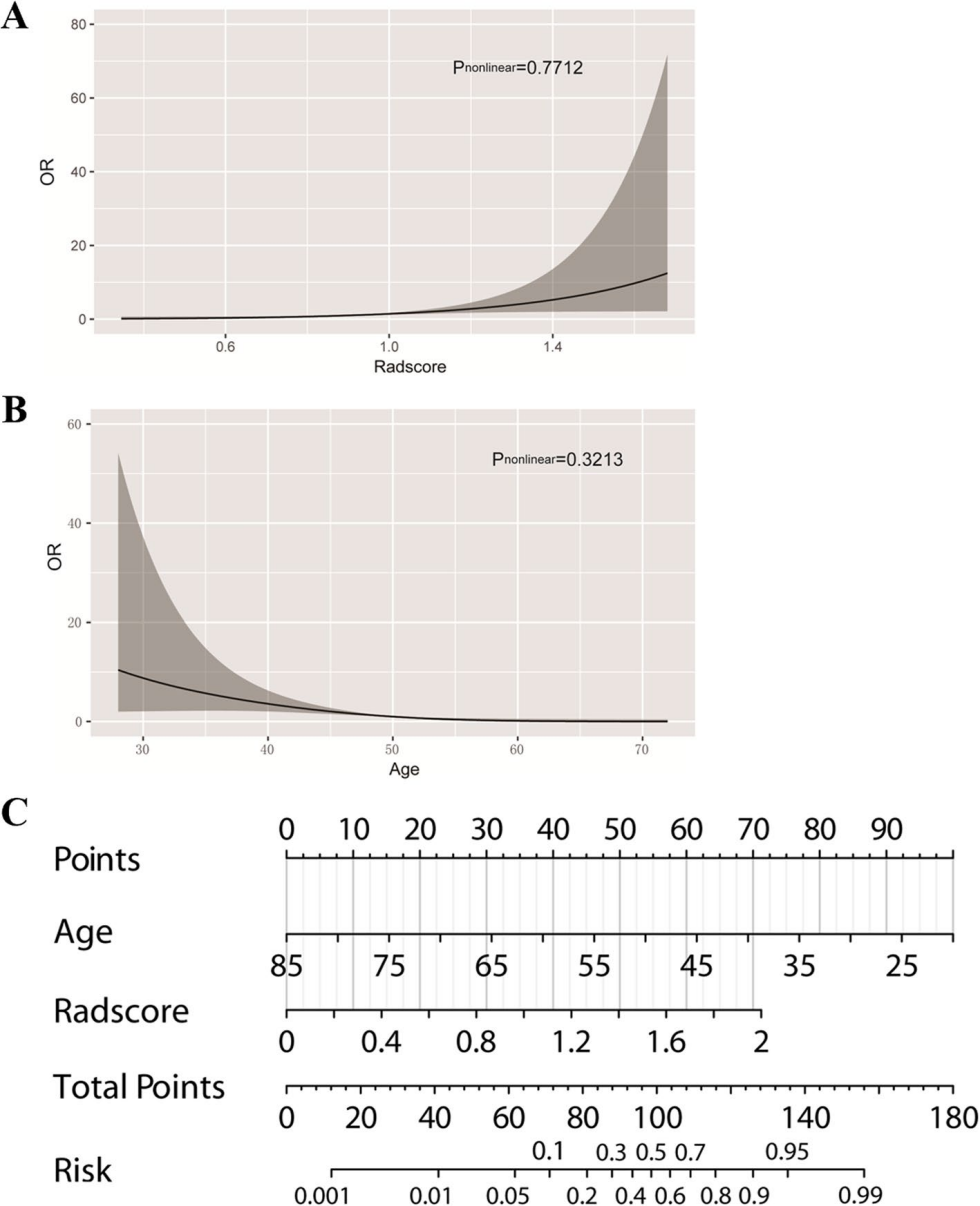
Model building. Fitting curves for the radscore (**A**) and age (**B**) are shown. In the visual nomogram (**C**), first, a vertical line was drawn according to the values of the most influential factors to determine the corresponding numbers of points. The total points were the sum of the above points. Then, a vertical line was drawn according to the value of total points to determine the probability of risk

In cohort 1, univariable analysis showed age, menstrual status, tumor location, CA125, CA125/CEA, CA724, CA19-9 and radscore were significantly associated with SOM. Next, a nomogram model was developed by multivariable logistic regression analysis of select risk factors (Age, OR = 0.884, *p* < 0.01; radscore, OR = 17.222, *p* = 0.001, Table 2 and Fig. 2A-B). The generated nomogram, depicted in Fig. 2C, had a higher AUC than the radscore and subjective evaluation (0.910 vs 0.827 vs 0.773) in the training set. The DeLong test revealed the differences were statistically significant (*p* = 0.007, *p* = 0.026). The bootstrap algorithm also showed a satisfactory performance for SOM, with an AUC of 0.907 in 1000 cross-validation, as an internal validation tool.

**Table 2:** Regression analysis for model building

	Negative (*n* = 63)	Positive (*n* = 38)	Univariable analysis		Multivariable analysis ^**a**^	
			OR (95% CI)	*P* value	OR (95% CI)	*P* value
**Menstrual status**				< 0.001	/	/
**postmenopause**	44(69.84)	4(10.53)	1 (reference)		/	/
**premenopausa**	19(30.16)	34(89.47)	19.684 (6.125, 63.258)		/	/
**Age (years)**	56.65 ± 11.99	38.34 ± 9.71	0.871 (0.826, 0.918)	< 0.001	0.884 (0.834, 0.937)	< 0.001
**Tumor location**				0.021	/	/
** U**	14(22.22)	6(15.79)	1 (reference)		/	/
**M**	19(30.16)	22(57.89)	2.702(0.867, 8.417)		/	/
**L**	30(47.62)	10(26.32)	0.778(0.236, 2.568)		/	/
**CEA**	2.000 (0.980, 3.410)	1.960 (1.170, 4.480)	1.007 (0.996, 1.019)	0.644	/	/
**CA125**	13.000 (9.000, 21.050)	61.400 (16.400, 162.500)	1.005 (1.000, 1.010)	< 0.001	/	/
**CA125/CEA**	6.856 (4.151, 17.185)	14.775 (5.939, 64.194)	1.010 (0.999,1.021)	0.004	/	/
**CA724**	1.920 (1.117, 4.370)	5.170 (3.640, 56.350)	1.013 (1.003, 1.023)	< 0.001	/	/
**CA19-9**	9.390 (4.380, 34.715)	29.100 (12.830, 186.400)	1.001 (1.000, 1.002)	0.011	/	/
**Radscore**	0.586 ± 0.274	0.995 ± 0.286	32.310 (7.771, 134.334)	< 0.001	17.222 (3.028, 97.958)	0.001

*OR* odds ratio, *U* upper third of the stomach, *M* middle third of the stomach, *L* lower third of the stomach

^a^stepwise regression

**Fig. 3:**
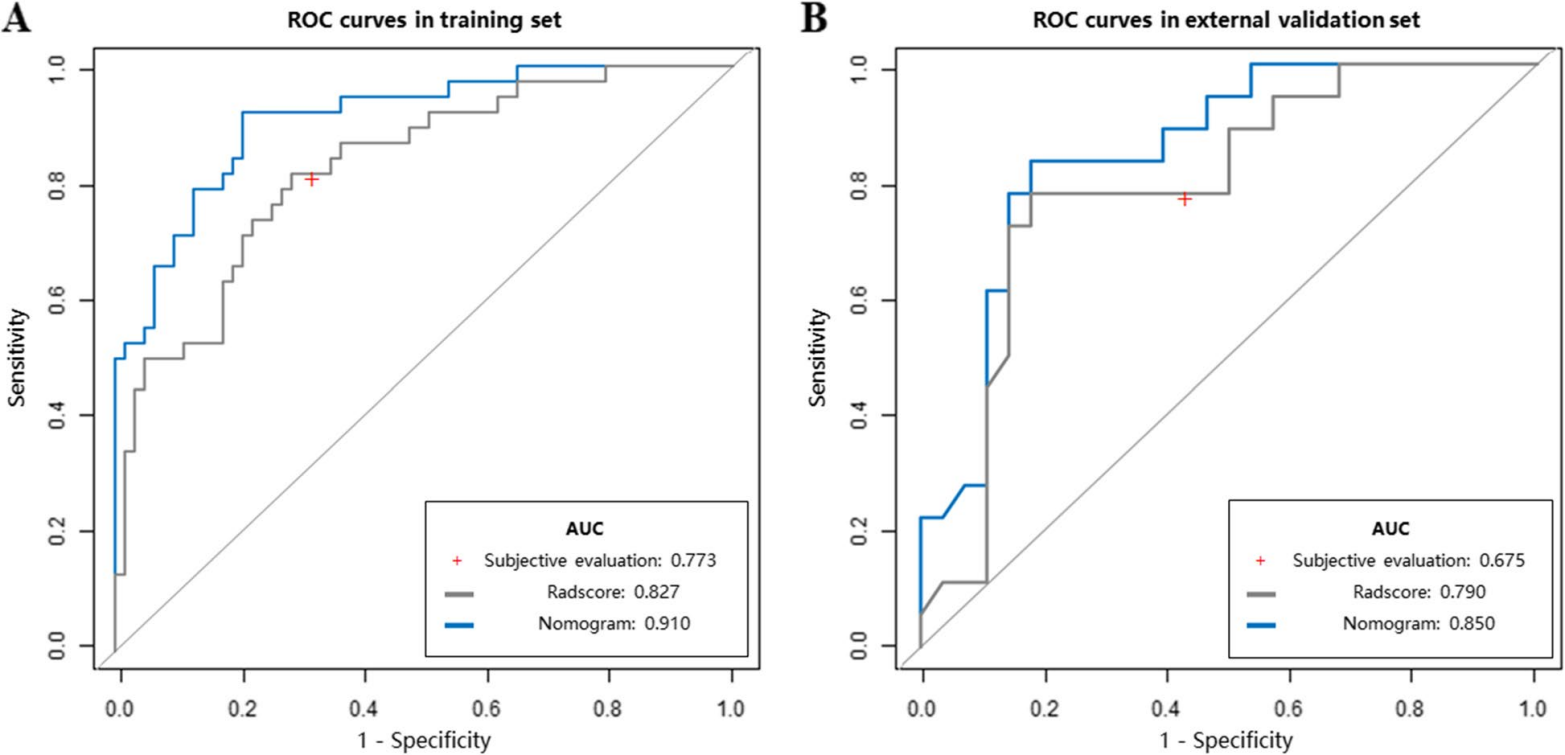
ROC curves in both data sets. **A** Training set. **B** External validation set

**Fig. 4:**
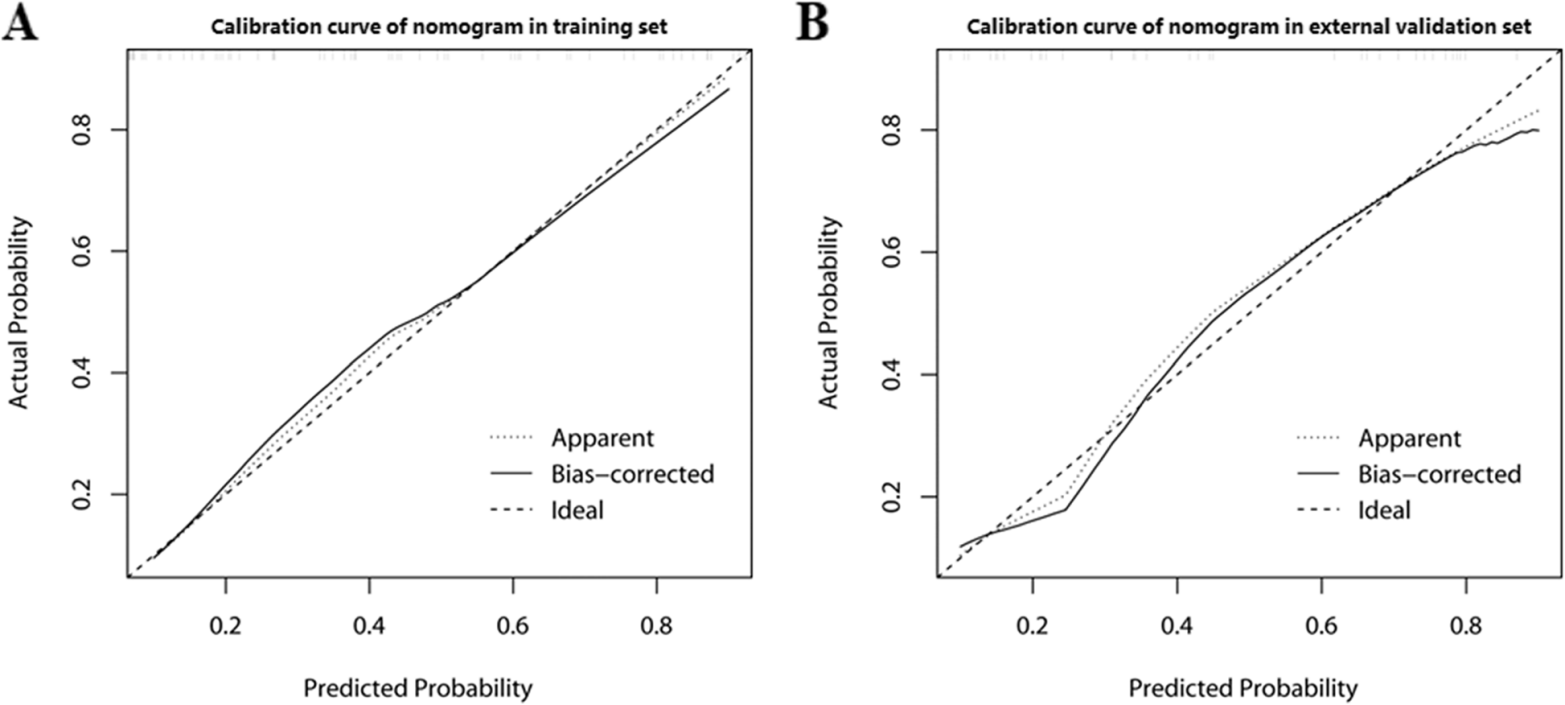
The calibration curves of the nomogram in both data sets. **A** Training set. **B** External validation set

In cohort 2 (external validation set), the nomogram model in combination with the radscore and age (details listed in Supplemental Table 2) had a higher AUC than the radscore and subjective evaluation (0.850 vs 0.790 vs 0.675). The detailed ROC analyses and comparisons are shown in Table 3 and Fig. 3. Calibration curves for the nomogram in both datasets suggested no significant deviation (Hosmer–Lemeshow test, all *P* > 0.05) from an ideal fitting (Fig. 4).

**Table 3:** ROC curve analysis and comparison of predictive values

	**Training set (*n* = 101)**			**External validation set (*n* = 46)**		
	**Nomogram**	**Radscore**	**Subjective evaluation**	**Nomogram**	**Radscore**	**Subjective evaluation**
**AUC**	0.910	0.827	0.773	0.850	0.790	0.675
**95% CI**	0.845–0.959	0.740–0.901	0.690–0.856	0.738–0.963	0.652–0.927	0.539–0.810
**Specificity**	0.794	0.714	0.730	0.821	0.821	0.571
**Sensitivity**	0.921	0.816	0.816	0.833	0.778	0.778
**Accuracy**	0.842	0.752	0.762	0.826	0.804	0.652
**PLR**	4.464	2.855	3.023	4.667	4.356	1.815
**NLR**	0.099	0.258	0.252	0.203	0.270	0.389
**PPV**	0.729	0.633	0.646	0.750	0.737	0.538
**NPV**	0.943	0.865	0.868	0.885	0.852	0.800
**Delong test (*P* value)** ^*^	/	0.007	0.026	/	0.186	0.047
**NRI** ^a^	/	-0.185	-0.169	/	-0.056	-0.306

*AUC* area under the curve, *PLR* positive likelihood ratio, *NLR* negative likelihood ratio, *NPV* negative predictive value, *PPV* positive predictive value, *NRI* net reclassification index

^*^Compared with nomogram model

**Fig. 5:**
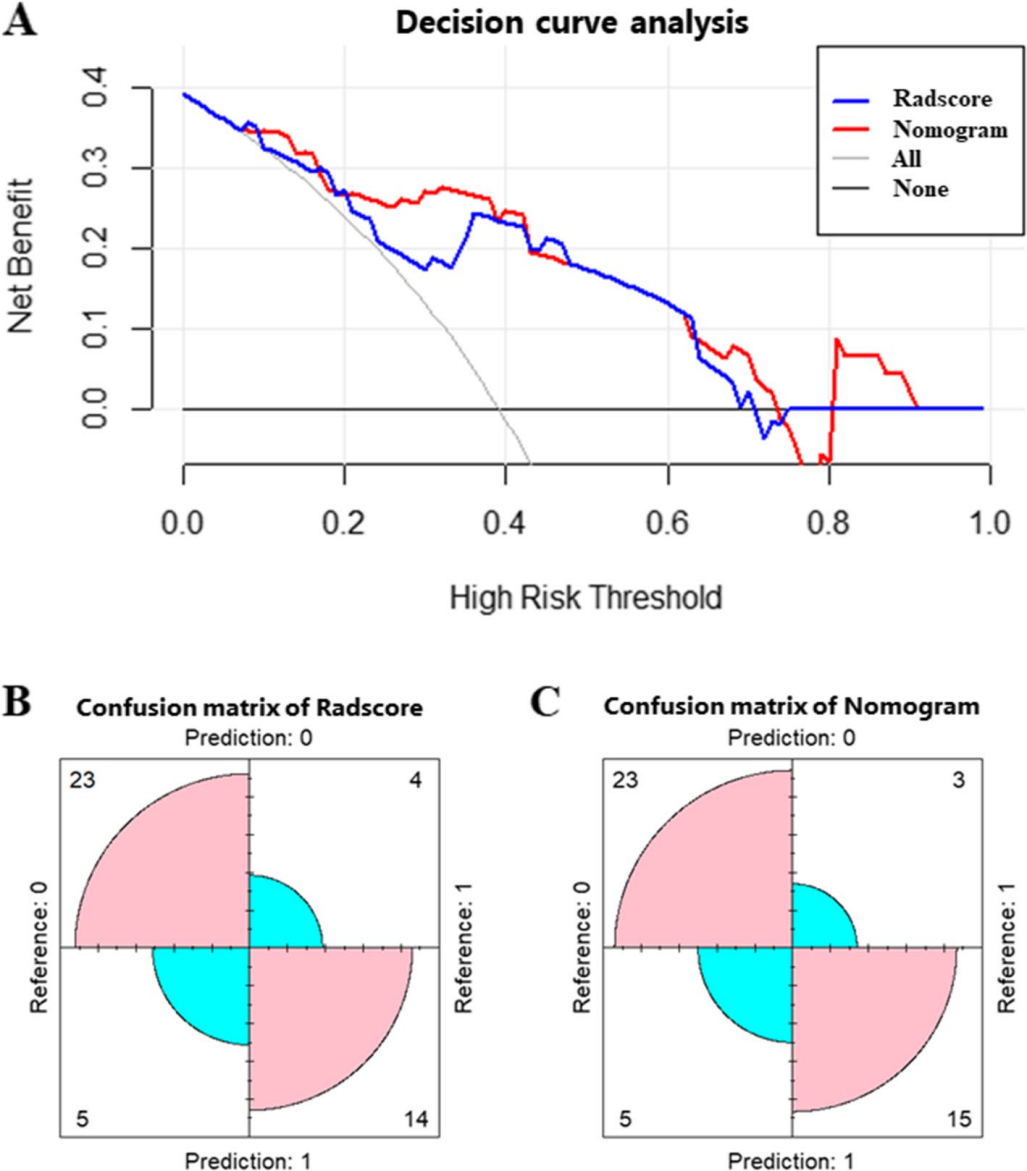
Model validation. **A** DCA in the external validation set. Light- and dark-grey lines represent the assumptions that all and no cases have a high risk, respectively. Red and blue curves showed that with a large probability range, utilizing the developed nomogram to predict the odds of SOM conferred a positive net benefit versus the radscore and the all-or-none scheme. The confusion matrix showed that using the nomogram model (**C**) would be more beneficial than applying the radscore alone (**B**)

In the external validation set, DCA showed that employing the novel nomogram to predict the probability of SOM conferred a positive net benefit compared to the radscore and the all-or-none scheme at a threshold probability from 10 to 75% (Fig. 5A). The confusion matrix confirmed the nomogram’s superiority over the radscore model (Fig. 5B-C).

## Discussion

This work developed a radiomics nomogram using CT and clinical data, which showed markedly enhanced power versus the radscore and subjective evaluation in detecting SOM in female GC patients. Clinicians can use this effective, noninvasive radiomics approach to improve the screening accuracy of SOM preoperatively and to design individuated treatments.

Detection of SOM in GC patients is important for clinical decision-making. Ovarian metastasis (OM) reduces prognosis in female GC cases and often results in failed treatment [26]. Despite recent advances in diagnostic and therapeutic tools for GC, GC cases with OM still show unsatisfactory prognosis, with median survival time of less than 15 months [7]. When SOM is diagnosed, the GC case has stage IV disease (cM1), with poor prognosis. Such patients are not eligible for curative surgery, and the treatment strategy may mainly include systemic therapy and chemoradiation, with the treatment goals being symptom relief and delayed progression. If a GC patient is diagnosed with primary ovary neoplasms, GC treatment is not affected, and treatment of ovary neoplasms may be evaluated by an obstetrician-gynecologist.

Timely detection and precise evaluation of SOM is fundamental to optimal therapeutic decision-making. According to the NCCN guidelines for gastric cancer (version 2.2022) [2], pelvis imaging evaluation should be performed for newly diagnosed patients for a thorough assessment. However, the imaging appearances of ovarian metastasis and primary ovarian neoplasms may overlap, and the correct diagnosis can be challenging for radiologists [27]. Typically, ovarian metastases mostly occur in premenopausal women and present as bilateral masses. Peritoneal metastasis could also be observed in multiple patients simultaneously. These features as well as the medical history are key clues for the diagnosis of ovarian metastasis. However, in a retrospective study, Lin et al. [8] reported that 29.2% of ovarian metastases were unilateral and 31.5% showed no peritoneal metastasis. When presented atypically, OM might be misinterpreted as a primary ovarian neoplasm or even physiologic ovarian enlargement. In a study by de Waal et al., about 25% of ovarian metastases mimicked a primary ovarian tumor [16]. Feng et al. [15] reported that 6 of 63 patients had erroneous diagnosis as physiologic ovarian enlargement by imaging modalities and received no timely treatment.

Many studies have assessed the clinicopathological characteristics and prognostic factors of OM; however, imaging diagnosis of SOM sometimes remains challenging for radiologists. Routine imaging modalities, including CT, ultrasound and MRI, are unsatisfactory in discriminating between primary and secondary ovary tumors. Because the traditional imaging features of SOM are non-specific and may lead to misdiagnosis, such evaluation is obviously a subjective process that lacks reliability. Therefore, the above facts highlight the urgent need to develop reliable tools to correctly identify GC cases with SOM and to improve prognosis.

Radiomics constitutes a new strategy using routine imaging findings to perform a high-throughput quantitative evaluation. This quantitative method provides a noninvasive tool for a more comprehensive assessment of the biological properties and heterogeneity of GC compared with morphological visual representation. It is widely admitted radiomics can be applied in GC evaluation [19, 21, 23, 28–30]. However, radiomics is scarcely applied for detecting SOM in GC.

Importantly, this study relied on the application of radiomics features derived from primary lesions. Though the routine use of pelvic CT/MRI in clinic could help detect ovarian masses, the imaging features of ovarian metastases sometimes can be atypical and misleading. This problem is aggravated by the lack of consensus on appropriate morphological criteria to assess OM involvement accurately. Compared to routine approaches, the radiomics approach is convenient, inexpensive, and free from risk of secondary inspection. The radiomics features extracted from primary tumor could directly predict the presence of SOM, no matter whether the ovarian region has benign lesions. Therefore, it could be utilized easily for identifying low-risk patients who may not benefit from further radical imaging examination, such as PET/CT, which may reduce the radiation exposure and save expenses.

It is noteworthy that we built a nomogram combining age and CT-based radscore, which constitutes a visualization tool with improved discriminatory ability for SOM detection. A nomogram was generated to help radiologists and clinicians assess SOM more easily. This model showed favorable performance and better diagnostic efficiency than subjective evaluation and the radscore alone. The AUC improved from 0.675 to 0.850, with a pretty higher sensitivity and accuracy of 0.833 and 0.826 in the validation cohort. Thus, the proposed nomogram could be clinically applied to promote risk stratification in patients with GC.

Another valuable aspect of the present work that an actual external validation cohort was examined. The external validation set in the current study revealed improved diagnostic performance and better clinical benefit with the use of the novel nomogram. This indicates utilizing an external set may help overcome the shortcoming of overfitting for a newly built model. Therefore, the new model may assist radiologists in improving diagnostic confidence and provide clinicians with more useful and objective understanding of overall prognostic factors beforehand in the clinical setting.

This study had limitations. First, it had a small sample and a retrospective design, indicating potential selection bias and reduced data generalizability. Therefore, larger multicenter studies with external validation cohorts are needed to address these shortcomings. In addition, imaging segmentation was performed manually rather than semi-automatically or automatically, which may result in subjective errors not suitable for large data processing. Compared to routine manual approach which often insufficiently systematic and cumbersome procedure, the deep learning-based automatic segmentation may thus help alleviate this burden and can effectively improve research reliability [31]. Additionally, imaging segmentation was performed driven from primary GC. Although most methodologies advocate the use of volume of the whole primary tumor, only the radiomics features of the primary tumor were extracted and analyzed, and the features of the OMs themselves were not explored, which may result in incomplete observation data. This point attracted a great attention both in theoretical and application fields. Furthermore, deep learning tools were not developed and validated for the detection of SOM or even peritoneal metastasis [32]. This application of artificial intelligence method could be used to guide personalized treatment plans with the help of computerized tumor-level characterization [33]. Finally, this study did not include relevant molecular biological indicators. “Radiogenomics” includes the radiomics and genomics features represents an emerging prognostic approach [34], which should be addressed in future studies.

## Conclusions

In conclusion, using preoperative CT images, a quantitative radscore was built to determine the risk of SOM in GC patients. Then, a nomogram model combining the radscore and clinical characteristics could be applied to improve the clinical benefit versus the subjective evaluation and the radscore alone. This visual noninvasive nomogram approach could be clinically applied to promote risk stratification in GC.

## Supplementary Information

**Additional file 1: Supplemental Table 1.** Interobserver agreement in both study cohorts. **Supplemental Table 2.** Logistic regression analysis in cohort 2. **Supplemental Fig. 1.** LASSO algorithm for feature selection.

## Data Availability

The datasets used and/or analyzed during the current study are available from the corresponding author on reasonable request.

## Abbreviations

GC: Gastric cancer
SOM: Synchronous ovarian metastasis
ICC: Intraclass correlation coefficient
VOI: Volume of interest
LASSO: Least absolute shrinkage and selection operator
ROC: Receiver operating characteristic
AUC: Area under the roc curve
DCA: Decision curve analysis
