# Supplementary material for: Assessing synchronous ovarian metastasis in gastric cancer patients using a clinical-radiomics nomogram based on baseline abdominal contrast-enhanced CT: a two-center study
Source: Cancer Imaging. 2023 Jul 24;23:71. doi: 10.1186/s40644-023-00584-5 (PMC10367237; doi:10.1186/s40644-023-00584-5)
Supplement: Supplementary file 1 — Additional file 1: Supplemental Table 1. Interobserver agreement in both study cohorts. Supplemental Table 2. Logistic regression analysis in cohort 2. Supplemental Fig. 1. LASSO algorithm for feature selection. [file 40644_2023_584_MOESM1_ESM.docx]

**Supplementary Materials**

**Supplemental Table 1. Interobserver agreement in both study cohorts**

| **Dataset** | **Radiologist 1 - Radiologist 2**  ***Kappa* value (95% CI)** | **Radiologist 1 - Radiologist 3**  ***Kappa* value (95% CI)** | **Radiologist 2 - Radiologist 3**  ***Kappa* value (95% CI)** | **All Radiologists**  **ICC (95% CI)** |
| --- | --- | --- | --- | --- |
| **Cohort 1** | 0.778 (0.744 - 0.833) | 0.847 (0.754 - 0.907) | 0.889 (0.834 - 0.942) | 0.853 (0.767 - 0.894) |
| **Cohort 2** | 0.756 (0.698 - 0.845) | 0.760 (0.696 – 0.873) | 0.872 (0.727 - 0.959) | 0.838 (0.760 - 0.871) |

ICC, intraclass correlation coefficient

**Supplemental Table 2. Logistic regression analysis in cohort 2**

|  | | **Univariable analysis** | | **Multivariable analysis** | |
| --- | --- | --- | --- | --- | --- |
|  |  | **OR (95% CI)** | ***P* value** | **OR (95% CI)** | ***P* value** |
| **Menstrual status** |  |  | 0.057 | / | / |
|  | **postmenopause** | 1 (reference) |  |  |  |
|  | **premenopausa** | 3.680 (0.962, 14.076) |  |  |  |
| **Age (years)** |  | 0.956 (0.902, 1.012) | 0.123 | 0.910 (0.836, 0.991) | **0.030** |
| **Tumor location** |  |  | 0.340 | / | / |
|  | **U** | 1 (reference) |  |  |  |
|  | **M** | 4.727 (1.056, 21.154) |  |  |  |
|  | **L** | 1.600 (0.202, 12.695) |  |  |  |
| **CEA** |  | 0.998 (0.995, 1.002) | 0.402 | / | / |
| **CA125** |  | 0.999 (0.996, 1.002) | 0.671 | / | / |
| **CA125/CEA** |  | 0.975 (0.942,1.010) | 0.159 | / | / |
| **CA724** |  | 0.997 (0.988, 1.007) | 0.563 | / | / |
| **CA19-9** |  | 1.001 (0.999, 1.003) | 0.222 | / | / |
| **Radscore** |  | 52.426 (4.584, 599.589) | **<0.001** | 145.207 (7.900, 2669.083) | **<0.001** |

*OR*, odds ratio

U, upper third of the stomach. M, middle third of the stomach. L, lower third of the stomach.

**Supplemental Figure 1.** **LASSO algorithm for feature selection.**


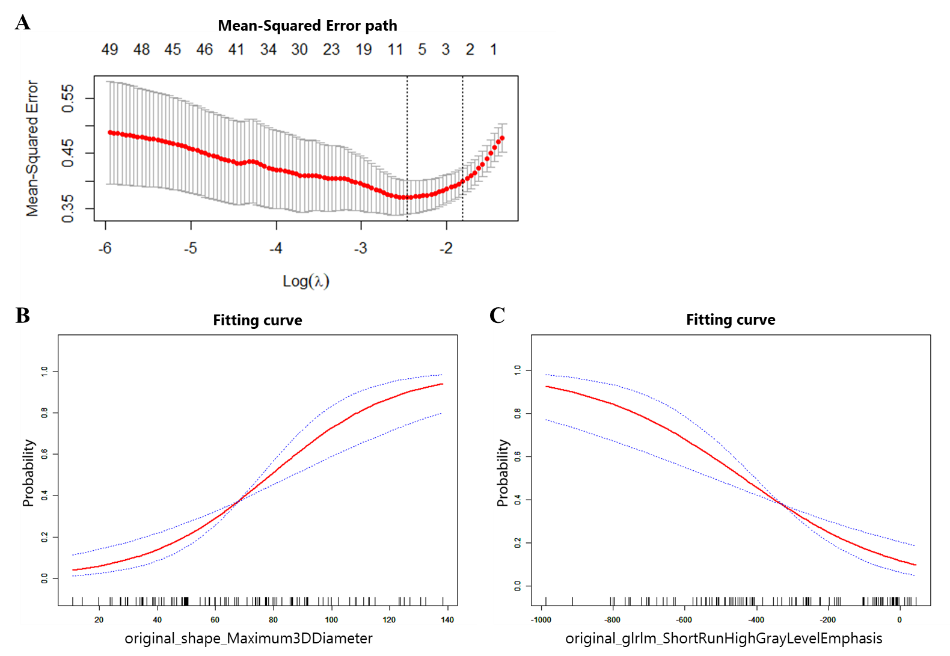


(A) Mean-Squared Error path. Ten-fold cross-validations for tuning parameter selection in the LASSO model. The solid vertical lines are deviance ± standard error (SE). The dotted vertical lines are drawn at the optimal values by minimum criteria and 1-SE criteria. We plotted the deviance versus log (λ), where λ is the tuning parameter. Herein, a value λ = 0.156 with log (λ) = -1.858 was chosen by 10-time cross-validations via 1-SE criteria. 2 features which are correspond to the optimal alpha value were selected. The fitting curves of the 2 final selected radiomics features: original_shape_Maximum3DDiameter (B) and original_glrlm_ShortRunHighGrayLevelEmphasis (C).
